# Removing Lead from Contaminated Sediment Using Indium-Based Perovskite Precursor

**DOI:** 10.3390/nano12244395

**Published:** 2022-12-09

**Authors:** Chen Tian, Zhenye Liang, Liwei Cheng, Shanglei Feng, Yiwen Li, Yingguo Yang, Lina Li

**Affiliations:** 1 Shanghai Synchrotron Radiation Facility (SSRF), Zhangjiang Lab., Shanghai Advanced Research Institute & Shanghai Institute of Applied Physics, Chinese Academy of Sciences, Shanghai 201204, China; 2 University of Chinese Academy of Sciences, Beijing 100049, China; 3 School of Microelectronics, Fudan University, Shanghai 200433, China

**Keywords:** perovskite, Pb replacement, in situ, synchrotron-based radiation experiment, GIWAXS

## Abstract

Heavy metal pollution in river and lake sediments seriously damages river ecological safety and indirectly affects human health. The existing research mainly focuses on how to adsorb pollutants and repair sediment, and how the reuse of these pollutants may be a new technology to control sediment pollutants. The rapid development of perovskite solar cells in recent years has attracted a lot of attention, among which lead (Pb) halide perovskites have very excellent photoelectric performance. In this study, we propose a novel idea of introducing indium (In)-based perovskite to replace Pb (II) ions dispersed in river and lake sediment. Three sediment samples from a river in Shanghai Peace Park were collected to analyze the distribution of heavy metal Pb. We mixed the digestion solution of sediment with the prepared CH_3_NH_3_(MA)InICl_2_ solution and found that indium (In) in perovskite precursor solution would be gradually replaced by Pb in sediments. An in situ synchrotron radiation XRD experiment was performed to reveal the reaction mechanism of solutions and provide a good research platform for the comprehensive reuse of sediment in the future. This study provides a new method of remediation of heavy metal pollution in river and lake sediments.

## 1. Introduction

With the vigorous development of science, technology and economy, it is inevitable that some pollutants, such as factory sewage and domestic waste, will be discharged into bodies of water [1,2]. After long-term accumulation, these pollutants are finally deposited in the sediments and continuously enriched. On the one hand, the sediments can adsorb the pollutants that are difficult to degrade in water; on the other hand, they will become a new pollution source when the adsorption is too much, which will cause a greater impact on the water [3,4]. The pollutants in sediment are mainly divided into three categories: heavy metals (such as Pb, arsenic (As), cadmium (Cd), mercury (Hg), copper (Cu), etc.), refractory organic matter, and nutrient elements (N, P, etc.) [5]. Heavy metals mainly come from rock weathering, industrial sewage discharge and domestic waste discharge [6]. Over time, heavy metals will gradually adsorb and precipitate on minerals and organic matter and eventually accumulate in river sediments, reaching a dynamic equilibrium in the aqueous phase. Most heavy metals are stored in sediment in various forms because of their toxicity and non-biodegradability of organic matter [7]. The accumulation of heavy metals in sediment will cause harm to fish and shrimp in the water environment and thus harm human health through the food chain. Moreover, sediments will gradually poison the food chain by releasing the stored heavy metals into the above water, biological enrichment or other secondary pollution channels [8,9]. Therefore, harmless and resource-based reuse of sediment will be a direction of future research.

At present, the commonly used remediation methods for sediment heavy metal pollution are in situ remediation and ectopic remediation, including sediment-masking technology [10,11], chemical-leaching technology [12], phytoremediation technology [13,14], microbial remediation technology [15], solidification/stabilization technology [16], etc. Sediment-masking technology and solidification/stabilization technology are methods of stabilizing sediment through physical–chemical methods and preventing the transfer of pollutants to water [17]. Chemical-leaching technology is a method which chemical extractants are added to the heavy metals leaching out from the sediment [18]. Phytoremediation technology and microbial remediation technology use organisms to remove heavy metals from soil and sediment [19]. Pb is one of the three major heavy metal pollutants in the environment. It is a heavy metal element which seriously harms human health. Long-term ingestion of Pb can cause serious damage to the blood and nervous system, especially in children [20]. Therefore, it is urgent to repair and remove heavy metals from sediment. Electrodialytic remediation experiments (EDR) [21,22] have become an effective method for restoring heavy metal Pb and other metals. Gunvor M. Kirkelund et al. [22] used electrodialytic remediation technology to remove heavy metals from sediment under oxic conditions. They found that the removal rate of metal was mainly dependent on the stirring velocity of the sediment suspension, which directly affected the pH value of the obtained sediment, while the effect of current density was not significant. They adjusted the conditions and found that the removal rates of heavy metals from the restored suspension were 44% Cu, 65% Pb, 78% Zn and 98% Cd, respectively.

Sediment remediation technologies have made great progress in improving the pollution of many rivers and lakes. However, at present, many remediation technologies of heavy metal contaminated sediment are aimed at effectively removing the heavy metals in sediment [23,24,25]. Few articles have studied how to extract heavy metals and reuse them. In recent years, lead-based perovskite solar cells (PSCs) have attracted extensive attention due to their excellent photoelectric characteristics [26,27,28,29,30] Ligang Wang et al. [31] introduced europium ion pairs (Eu^3+^−Eu^2+^) into perovskite precursor solutions and films to promote the conversion of Pb^0^ and I^0^ into Pb^2+^ and I^−^, causing perovskite films to have higher long-term stability. Zhaokui Wang et al. [32] synthesized mixed organic-inorganic perovskite MAPb_1–*x*_In*_x_*I_3_Cl_x_ by replacing Pb with part indium (In). Grazing incidence X-ray diffraction (GIXRD) results show that Pb–In perovskites have higher crystallinity and multiple ordered crystal orientations, which make Pb–In planar heterojunction devices have higher efficiency than purely Pb-based devices. This changes the form of heavy metals from toxic to non-toxic through physical or chemical reactions and significantly improves their stability. If Pb in sediment can be used in perovskite solar cells, they may be a new way of removing heavy metal pollution from rivers.

In this work, inspired by the above research, we synthesized MAInICl_2_ perovskite precursor solution and obtained the digestion solution through microwave digestion of sediment samples. We mixed the digested solution of the precipitate with the prepared MAInICl_2_ solution in different proportions, and found that the mixed solution had a complex reaction; we guessed that part of indium (In) in the perovskite precursor solution would be gradually replaced by Pb in the precipitate. In addition, we designed a simple liquid reaction cell and observed in situ the reaction process during the mixing of digestion solution and perovskite precursor solution by using a high-resolution synchrotron radiation XRD method. This work evaluated the possibility that Pb from river sediments could be reused in perovskite materials. However, due to the uncertainty of the river environment and the complex composition of the sediment, further research is needed in the future; this could expand the sampling site and select appropriate sediment samples. This study provides a new idea for the remediation of heavy metal pollution in river and lake sediments.

## 2. Results

We randomly selected three sampling sites in Shanghai Peace Park, collected three river sediment samples with a stainles steel grab sampler and then stored the samples in a shade bag at low temperature. After the samples were dried in a cool and ventilated place for a week, the sediment samples were digested by microwave, and the content of heavy metal Pb in the digestion solution was analyzed by inductively coupled plasma-mass spectrometry (ICP-MS). The concentrations of Pb were 45.302, 40.611 and 46.154 mg/kg, respectively. The digestion solution with high Pb content was mixed with perovskite precursor solution.

**Figure 1:**
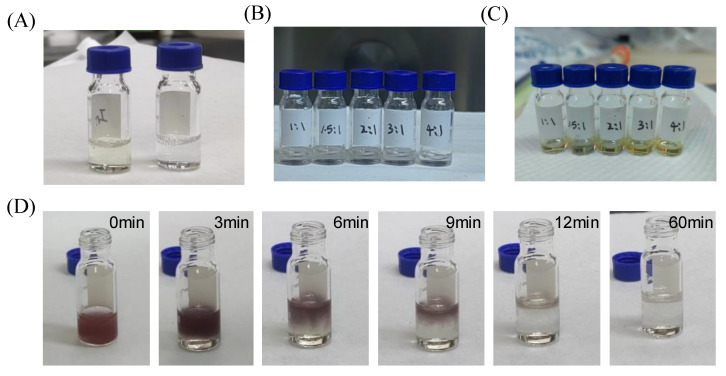
(**A**) Image of MAInICl_2_ perovskite precursor solution (**left**) and the digestion solution (**right**). Images of the mixture of perovskite precursor solution and digestion solution in different proportions on (**B**) day 1 and (**C**) day 3. (**D**) Images of precipitation change process of perovskite precursor solution mixed with digestion solution.

The MAInICl_2_ solution was mixed with the digestion solution in four ratios: 1:1, 1.5:1, 2:1 and 3:1. For ease of description, the samples with these four ratios are named 1-1, 3-2, 2-1 and 3-1. Figure 1A shows images of the yellowish MAInICl_2_ solution (left) and the colorless and transparent digestion solution (right). However, when the two solutions were mixed in different proportions, all five samples appeared colorless and transparent on the first day (Figure 1B). After 3 days of placement, the solution changed from colorless to pale yellow, as shown in Figure 1C. This suggests that in addition to the obvious reaction that occurred at the beginning when the solution was mixed, slow chemical reactions took place over the next few days. After leaving the mixture for a few more days, the color of the solution did not change significantly. In addition, when mixing the two solutions, it was found that a large number of purple precipitates first appeared in the mixed solution, which gradually disappeared into colorless and transparent solution within a few minutes, as shown in Figure 1D. Additionally, the higher the proportion of the digestion solution, the slower the precipitation disappeared.

**Figure 2:**
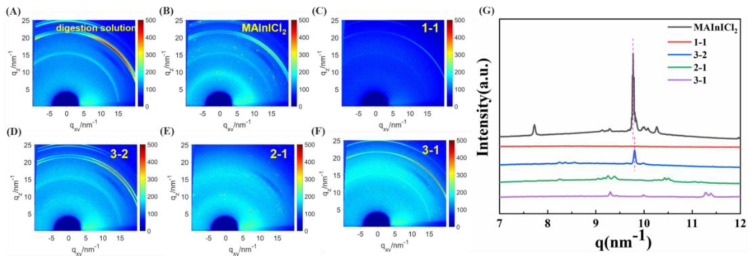
(**A**–**G**) 2D-GIWAXS images and the corresponding integrated curves of the film of the digestion solution, MAInICl_2_ and mixtures in different proportions.

In order to study the changes of the mixture of two solutions, perovskite precursor solution, digestion solution and different proportions of mixed solutions left for a few days were coated on ITO glass substrates and annealed to test the GIWAXS results of the film. The grazing incidence angle used in the test is 0.4°. Figure 2A–G show the two-dimensional (2D) grazing incidence wide-angle X-ray scattering (GIWAXS) plots of several films and the corresponding integral curves. It can be seen that there are several bright diffraction rings in the 2D images of these films, which can be considered ITO signals on the substrate. The ITO signals are slightly different due to the different placement of samples during the test. Perovskite film has a typical diffraction ring at q ≈ 10 nm^−1^, where q is the scattering vector corresponding to (110) crystal planes [33,34]. Different proportions of mixed solution of thin film signal were also slightly different; only the film with a ratio of 3-2 had the same q ≈ 10 nm^−1^ signal. In addition, the position of the diffraction peak is red shifted, the width of the half-peak is narrowed and there is no splitting peak. It can be assumed that the Pb in the digestion solution replaced part of In of the perovskite precursor solution; this would agree with the previous literature [32]. However, there is no similar diffraction ring signal in other proportions of mixed solution of thin film, which also indicates that Pb can replace part of In only when the digestion solution and perovskite precursor solution are mixed in appropriate proportions. In addition, the SEM test and EDX analysis of the film of 3-2 samples are shown in Figure S1. The surface of the film showed some patterns, indicating that the perovskite crystallization was not very good; this was consistent with GIWAXS results that the crystallinity of the peak at q ≈ 10 nm^−1^ of the film of the 3-2 sample was lower than that of the MAPbICl_2_ film. In the EDX analysis, we found that elements In, Pb and I are distributed uniformly on the film, while element Cl is not distributed uniformly. At the same time, we also found that the content of Pb in the film is lower than In element. This indicates that a small amount of Pb replaces In in perovskite.

**Figure 3:**
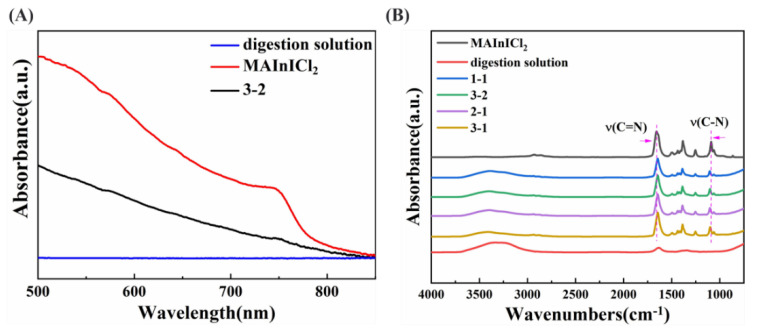
(**A**) UV-vis spectra of MAInICl_2_ perovskite precursor solutions, digestion solution and mixtures (3-2). (**B**) IR spectra of MAInICl_2_ perovskite precursor solutions, digestion solution and mixtures in different proportions.

Figure 3A shows the UV-vis absorption spectra of MAInICl_2_ perovskite precursor solutions, digestion solution and mixtures’ (3-2) films on ITO substrates. There is no obvious UV absorption in the digestion solution sample; the MAInICl_2_ perovskite film has obvious UV absorption of about 750 nm, and the mixed solution sample has relatively weak UV absorption at the same position. This also indicates that little In was replaced in the mixed solution; therefore, the position of the absorption peak did not change significantly. In order to further investigate the changes of the two solutions before and after mixing, the infrared (IR) spectra of the two solutions and the mixture after several days of placement were tested (Figure 3B). Attenuated total reflection (ATR) mode was selected as the test mode as it is more suitable for solution samples and requires less sample size. For the test, the solution was dropped onto an ATR crystal reflector and the liquid was touched with a pressure rod. The IR spectra of the samples were measured with 64 scans separately. In Figure 3B, the digestion solution has obvious water signal at 3400 cm^−1^, while the perovskite precursor solution has an obvious absorption peak at 1655 cm^−1^ and 1089 cm^−1^, which can be attributed to the C=N and C−N in the solution. The mixed solution contains the signature infrared peak of the two solutions; it was found that the infrared peak of C=N in the mixed solution had a red shift compared with the perovskite precursor solution, and the infrared peak of C−N had a blue shift. This indicates that the digestion solution interacted with perovskite precursor solution after mixing, and it is speculated that Pb partially replaced In in perovskite, leading to a change in chemical environment.

**Figure 4:**
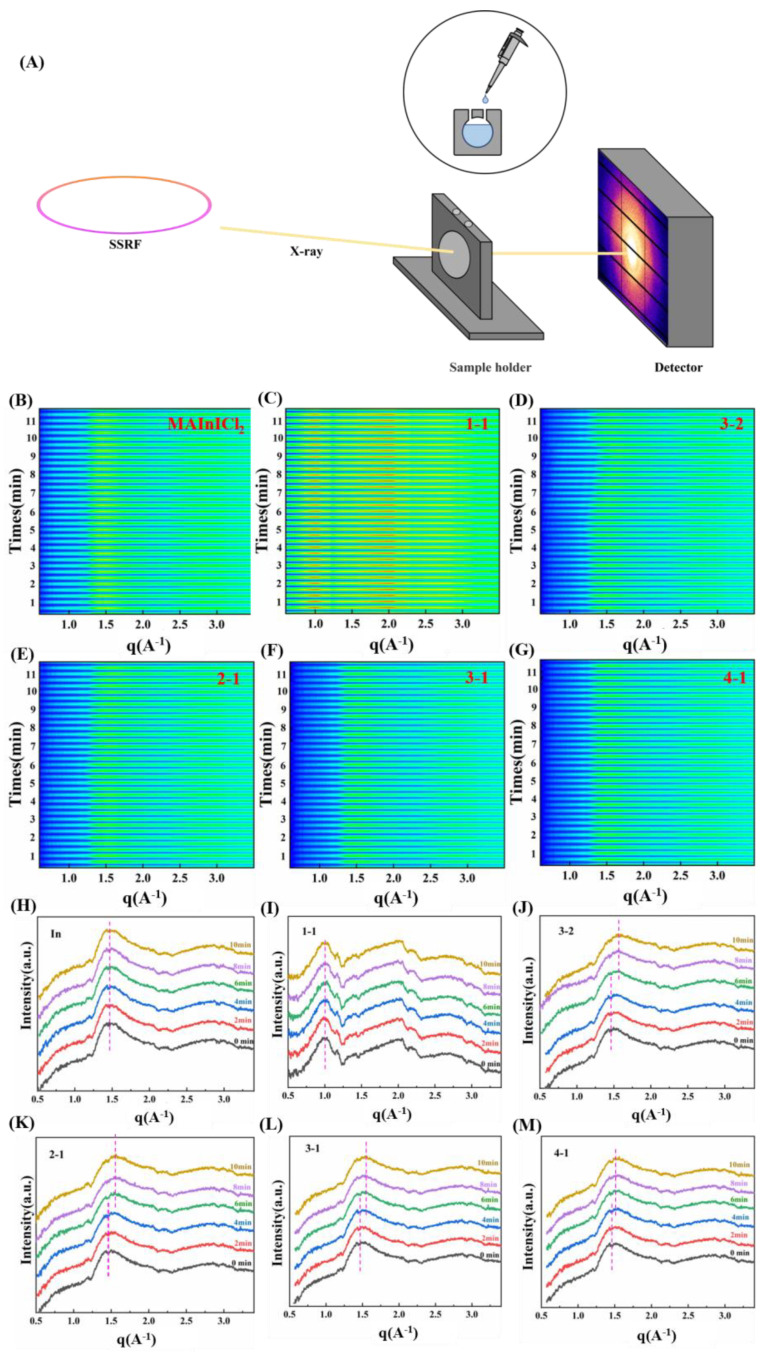
In situ GIWAXS measurements of MAInICl_2_ perovskite precursor solution and different ratios of MAInICl_2_ perovskite precursor solution mixed with digestion solution. (**A**) Diagram of GIWAXS experimental setup for in situ testing liquid samples. (**B**–**G**) Contour plots of integrated 1D-GIWAXS spectra of MAInICl_2_ perovskite precursor solution and mixed solutions of different proportions (each data interval is 20 s). (**H**–**M**) Images of 1D-GIWAXS integral spectra of MAInICl_2_ perovskite precursor solution and mixed solutions of different proportions at six time points (0 min, 2 min, 4 min, 6 min, 8 min and 10 min).

In order to further study the reaction between perovskite precursor solution and digestion solution at the beginning of mixing, we designed a simple liquid reaction tank, as shown in Figure 4A. There is a hollow hole in the middle of the liquid cell, and two tiny channels above it for injecting solution. Kapton tape was placed on both sides of the liquid pool to seal the large hole in the middle. The large hole in the liquid cell can hold a maximum of about 100 μL of liquid. During the test, the digestion solution was first added to the liquid cell with a needle, then the liquid cell was fixed to the experimental bench and the position was adjusted so that the X-ray could just pass through the large hole in the middle. A certain amount of MAInICl_2_ perovskite precursor solution was then dripped through a separate orifice above before preparation for the test. The GIWAXS experiment was carried out at BL17B1 beamline station of Shanghai Synchrotron Radiation Facility (SSRF). The selected X-ray energy was 10 KeV and the corresponding wavelength was 0.124 nm. The standard sample used is lanthanum hexaboride (LaB_6_), which is used to calibrate the distance between the sample, the detector and the position of the center of the circle. Due to the advantages of synchrotron radiation, such as high resolution and high throughput, the time needed to test a sample is in the order of seconds. Therefore, after mixing the samples, we selected 20 s to collect one datum, and each sample collected 34 data continuously, which took about 11–12 min. The integrated in situ 1D-GIWAXS spectra of MAInICl_2_ perovskite precursor solutions and mixtures of different proportions are shown in Figure 4B–G. In order to clearly show the changing process of the mixed solution, six time points were selected and drawn into a line chart, as shown in Figure 4H–M. The perovskite precursor solution had a wide peak at about q ≈ 1.5 A^−1^, which can be attributed to the precursor phase of the MAInICl_2_ solution. It remained stable for more than 10 min under X-ray irradiation, and the position and intensity of the peak were basically unchanged. The intensity of the peak in the samples of 3-2, 2-1, 3-1 and 4-1 does not change much in Figure 4J–M. With the increase of time, the broad peak at q ≈ 1.5 A^−1^ gradually shifted to the high angle direction, and the peak width gradually increased, which is speculated to be caused by the replacement of In by Pb. Additionally, the higher the proportion of digestion solution, the greater the peak offset angle. This is due to the fact that the larger the amount of digestion solution in the mixed solution, the longer the reaction time; this is consistent with the phenomenon that the purple precipitation of the 3-2 sample in the mixed solution had a longer retention time. Interestingly, the spectra of the 1-1 sample were quite different from those of the other four samples. The peaks of the 1-1 sample mainly appear at q ≈ 1.0 A^−1^ and q ≈ 2.0 A^−1^ in Figure 4I, and the intensity of the two peaks is stronger than that of samples with other proportions. However, with the increase in time, the positions of the peaks do not change significantly. It is speculated that the two peaks are mainly the liquid phase of excessive digestion solution, and the precursor phase of perovskite is covered because of the weak signal. Therefore, the Pb in the digestion solution can be successfully replaced by In in the perovskite precursor solution by choosing the appropriate proportion of mixed solution, which provides the possibility of synthesis of new perovskite materials.

## 3. Conclusions

In conclusion, it was found that Pb in the digestion solution of sediment can substitute In in the perovskite precursor solution. When the optimum mixing ratio is 1.5:1, the diffraction peak at q ≈ 10 nm^−1^ shifts and the half-peak width narrows (indicating that a proportion of Pb enters the perovskite), while other ratios do not have similar peaks. The in situ GIWAXS experiment observed the changes in the digestion solution and perovskite precursor solution about 10 min after the beginning of mixing, and found that the peak of the perovskite’s precursor phase shifted to a high angle direction, and the peak width gradually increased. This work simply proves the feasibility of using Pb in sediment to replace In in perovskite, and the development potential of Pb in perovskite materials. This work provides a significant new direction for the treatment and reuse of heavy metals in river and lake sediment and soil.

## 4. Materials and Methods

### 4.1. Materials

Methylamine iodide (MAI, 99.99%), indium trichloride (InCl_3_, 99.99%), N, N-dimethylformamide (DMF, anhydrous) were purchased from Sigma–Aldrich, Co., St. Louis, MO, USA.

### 4.2. Sample Collection and Analysis

Three superficial sediment samples (0–20 cm) were collected from rivers in Shanghai Peace Park. Sediments were collected with a stainless-steel grab sampler, sealed in polyethylene bags in dark places and stored at 5 °C and below. The sediment samples were dried at room temperature, then homogenized and sieved. An appropriate amount of the processed samples was digested with HNO_3_-HF mixture in a microwave, using appropriate digestion procedures. After cooling, the digested solution was filtered and diluted with deionized water to a suitable concentration. The concentration of lead was determined by ICP-MS.

### 4.3. Solution and Film Preparation

The CH_3_NH_3_InICl_2_ perovskite precursor solution was prepared by mixing the 159 mg MAI and 221 mg InCl_3_ in 1.5 mL DMF solvent in N_2_ glove box. Perovskite precursor solution and digestion solution were mixed in the following proportions: (1:1, 1.5:1, 2:1, 3:1). The CH_3_NH_3_InICl_2_ film was spin coated on the ITO substrate at 4000 rpm for 40 s in an N_2_ glovebox, and annealed from 60 to 100 °C at a ramp rate of 10 °C/5 min on a hot plate.

### 4.4. Characterization

Inductively coupled plasma-mass spectrometry (ICP-MS) results were obtained using Agilent 7800. UV-vis absorption spectra were acquired using a Shimadzu UV–2700 spectrophotometer over the 300–1000 nm wavelength range. The grazing incidence wide-angle X-ray scattering (GIWAXS) measurements were performed at the BL14B1 and BL17B1 beamline of the Shanghai Synchrotron Radiation Facility (SSRF), using X-ray with a wavelength of 0.6887 Å and 1.24 Å. Infrared spectra (IR) were examined on in attenuated total reflection (ATR) mode at BL01B1 beamline of the SSRF.

## Supplementary Materials

The following supporting information can be downloaded at: https://www.mdpi.com/article/10.3390/nano12244395/s1, Figure S1: (A) SEM images of film of 3-2 sample. (B–E) EDX elemental mapping of film of 3-2 sample.

## References

[1] Vanthuyne M., Maes A., Cauwenberg P. (2003). The use of flotation techniques in the remediation of heavy metal contaminated sediments and soils: An overview of controlling factors. Miner. Eng..

[2] Huang D., Liu L., Zeng G., Xu P., Huang C., Deng L., Wang R., Wan J. (2017). The effects of rice straw biochar on indigenous microbial community and enzymes activity in heavy metal-contaminated sediment. Chemosphere.

[3] Mohammed A.S., Kapri A., Goel R. (2011). Heavy metal pollution: Source, impact, and remedies. Biomanagement Met.-Contam. Soils.

[4] Gong J., Xie P. (2020). Research progress in sources, analytical methods, eco-environmental effects, and control measures of microplastics. Chemosphere.

[5] Yadav A., Chowdhary P., Kaithwas G., Bharagava R. (2017). Toxic metals in the environment: Threats on ecosystem and bioremediation approaches. Handb. Met.-Microbe Interact. Bioremediation.

[6] Bradl H. (2005). Sources and origins of heavy metals. Interface Sci. Technol..

[7] Sheoran A., Sheoran V. (2006). Heavy metal removal mechanism of acid mine drainage in w etlands: A critical review. Miner. Eng..

[8] Yunus K., Zuraidah M., John A.J.E., Change C. (2020). A review on the accumulation of heavy metals in coastal sediment of Peninsular Malaysia. Ecofeminism Clim. Chang..

[9] Ayas Z., Ekmekci G., Yerli S.V., Ozmen M. (2007). Heavy metal accumulation in water, sediments and fishes of Nallihan Bird Paradise, Turkey. J. Environ. Biol..

[10] Ke X., Bao Q., Qi Y., Huang X., Zhang H. (2018). Toxicity assessment of sediments from the Liaohe River Protected Area (China) under the influence of ammonia nitrogen, heavy metals and organic contaminants. Environ. Toxicol. Pharmacol..

[11] Costa-Böddeker S., Hoelzmann P., de Stigter H.C., van Gaever P., Huy H.Đ., Schwalb A. (2018). The hidden threat of heavy metal pollution in high sedimentation and highly dynamic environment: Assessment of metal accumulation rates in the Thi Vai Estuary, Southern Vietnam. Environ. Pollut..

[12] Mulligan C.N., Yong R.N., Gibbs B.F., James S., Bennett H. (1999). Metal removal from contaminated soil and sediments by the biosurfactant surfactin. Environ. Sci. Technol..

[13] Sakakibara M., Ohmori Y., Ha N.T.H., Sano S., Sera K. (2011). Phytoremediation of heavy metal-contaminated water and sediment by Eleocharis acicularis. Clean Soil Air Water.

[14] Cunningham S.D., Lee C.R. (1995). Phytoremediation: Plant-based remediation of contaminated soils and sediments. Bioremediation: Sci. Appl..

[15] Chen S.Y., Lin J.G. (2004). Bioleaching of heavy metals from contaminated sediment by indigenous sulfur-oxidizing bacteria in an air-lift bioreactor: Effects of sulfur concentration. Water Res..

[16] Gougar M., Scheetz B., Roy D. (1996). Ettringite and C−S−H Portland cement phases for waste ion immobilization: A review. Waste Manag..

[17] Higgins T.E., Halloran A.R., Petura J.C. (1997). Traditional and innovative treatment methods for Cr (VI) in soil. J. Soil Contam..

[18] Wang F., Yu J., Xiong W., Xu Y., Chi R. (2018). A two-step leaching method designed based on chemical fraction distribution of the heavy metals for selective leaching of Cd, Zn, Cu, and Pb from metallurgical sludge. Environ. Sci. Pollut. Res..

[19] Thakare M., Sarma H., Datar S., Roy A., Pawar P., Gupta K., Pandit S., Prasad R. (2021). Understanding the holistic approach to plant-microbe remediation technologies for removing heavy metals and radionuclides from soil. Curr. Res. Biotechnol..

[20] Finkelstein Y., Markowitz M.E., Rosen J.F. (1998). Low-level lead-induced neurotoxicity in children: An update on central nervous system effects. Brain Res. Rev..

[21] Pedersen K.B., Kirkelund G.M., Ottosen L.M., Jensen P.E., Lejon T.J. (2015). Multivariate methods for evaluating the efficiency of electrodialytic removal of heavy metals from polluted harbour sediments. J. Hazard. Mater..

[22] Kirkelund G.M., Ottosen L.M., Villumsen A. (2009). Electrodialytic remediation of harbour sediment in suspension—Evaluation of effects induced by changes in stirring velocity and current density on heavy metal removal and pH. J. Hazard. Mater..

[23] Dahrazma B., Mulligan C. (2007). Investigation of the removal of heavy metals from sediments using rhamnolipid in a continuous flow configuration. Chemosphere.

[24] Khan S., Ahmad I., Shah M.T., Rehman S., Khaliq A. (2009). Use of constructed wetland for the removal of heavy metals from industrial wastewater. J. Environ. Manag..

[25] Wen J., Yi Y., Zeng G. (2016). Effects of modified zeolite on the removal and stabilization of heavy metals in contaminated lake sediment using BCR sequential extraction. J. Environ. Manag..

[26] Yang Y., Lu H., Feng S., Yang L., Dong H., Wang J., Tian C., Li L., Lu H., Jeong J. (2021). Modulation of perovskite crystallization processes towards highly efficient and stable perovskite solar cells with MXene quantum dot-modified SnO_2_. Energy Environ. Sci..

[27] Jeong J., Kim M., Seo J., Lu H., Ahlawat P., Mishra A., Yang Y., Hope M.A., Eickemeyer F.T. (2021). Pseudo-halide anion engineering for α-FAPbI_3_ perovskite solar cells. Nature.

[28] Wang K., Yang Y., Lou Y., Li M., Igbari F., Cao J., Chen J., Yang W., Dong C., Li L. (2021). Smelting recrystallization of CsPbBrI_2_ perovskites for indoor and outdoor photovoltaics. eScience.

[29] Miao Y., Wang Y., Zhang H., Zhang T., Wei N., Liu X., Chen Y., Chen J., Zhao Y. (2021). In situ growth of atomic layer perovskitoid to stabilize and passivate MAPbI_3_ for efficient and stable photovoltaics. eScience.

[30] Wang S., Wang P., Chen B., Li R., Ren N., Li Y., Shi B., Huang Q., Zhao Y., Grätzel M. (2022). Suppressed recombination for monolithic inorganic perovskite/silicon tandem solar cells with an approximate efficiency of 23%. eScience.

[31] Wang L., Zhou H., Hu J., Huang B., Sun M., Dong B., Zheng G., Huang Y., Chen Y., Li L.J.S. (2019). A Eu^3+^–Eu^2+^ ion redox shuttle imparts operational durability to Pb-I perovskite solar cells. Science.

[32] Wang Z.K., Li M., Yang Y.G., Hu Y., Ma H., Gao X.Y., Liao L.S. (2016). High efficiency Pb–In binary metal perovskite solar cells. Adv. Mater..

[33] Feng S., Yang Y., Li M., Wang J., Cheng Z., Li J., Ji G., Yin G., Song F., Wang Z. (2016). High-Performance Perovskite Solar Cells Engineered by an Ammonia Modified Graphene Oxide Interfacial Layer. ACS Appl. Mater. Interfaces.

[34] Lu H., Liu Y., Ahlawat P., Mishra A., Tress W.R., Eickemeyer F.T., Yang Y., Fu F., Wang Z., Avalos C. (2020). Vapor-assisted deposition of highly efficient, stable black-phase FAPbI_3_ perovskite solar cells. Science.

