# Supplementary material for: Removing Lead from Contaminated Sediment Using Indium-Based Perovskite Precursor
Source: Nanomaterials (Basel). 2022 Dec 9;12(24):4395. doi: 10.3390/nano12244395 (PMC9783751; doi:10.3390/nano12244395)
Supplement: Supplementary file 1 [file nanomaterials-12-04395-s001.zip › nanomaterials-2044375-supplementary.pdf]

nanomaterials

Supplementary Materials

Removing Lead from Contaminated Sediment Using Indium-Based Perovskite Precursor

Chen Tian [1,2], Zhenye Liang [1,2], Liwei Cheng [1,2], Shanglei Feng [1,2], Yiwen Li [1,2], Yingguo Yang [1,2,3,*] and Lina Li [1,2,*]

[1] Shanghai Synchrotron Radiation Facility (SSRF), Zhangjiang Lab, Shanghai Advanced Research Institute, Shanghai Institute of Applied Physics, Chinese Academy of Sciences, Shanghai 201204, China
[2] University of Chinese Academy of Sciences, Beijing 100049, China
[3] School of Microelectronics, Fudan University, Shanghai 200433, China
* Correspondence: yangyingguo@sinap.ac.cn (Y.Y.); lilina@sinap.ac.cn (L.L.)

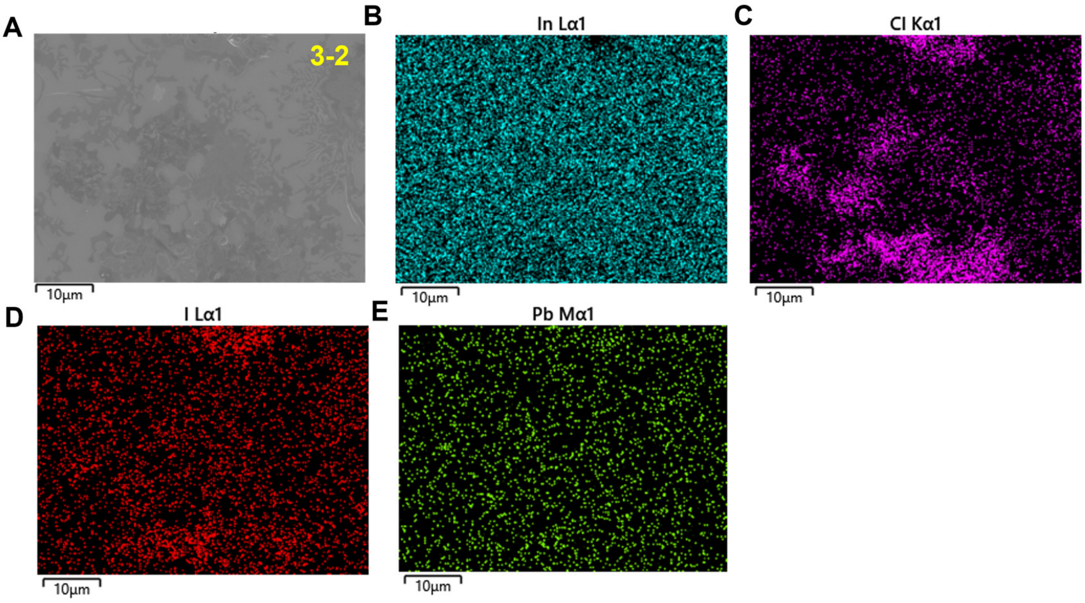

Figure S1. (**A**) SEM images of film of 3-2 sample. (**B–E**) EDX elemental mapping of film of 3-2 sample.
